# Interaction of Mycotoxin Alternariol with Serum Albumin

**DOI:** 10.3390/ijms20092352

**Published:** 2019-05-12

**Authors:** Eszter Fliszár-Nyúl, Beáta Lemli, Sándor Kunsági-Máté, Luca Dellafiora, Chiara Dall’Asta, Gabriele Cruciani, Gábor Pethő, Miklós Poór

**Affiliations:** 1 Department of Pharmacology, Faculty of Pharmacy, University of Pécs, Szigeti út 12, H-7642 Pécs; Hungary; eszter.nyul@aok.pte.hu (E.F.-N.); gabor.petho@aok.pte.hu (G.P.); 2 János Szentágothai Research Centre, University of Pécs, Ifjúság útja 20, H-7642 Pécs; Hungary; beata.lemli@aok.pte.hu (B.L.); kunsagi-mate.sandor@gytk.pte.hu (S.K.-M.); 3 Institute of Organic and Medicinal Chemistry, Medical School, University of Pécs, Szigeti út 12, H-7624 Pécs, Hungary; 4 Department of Pharmaceutical Chemistry, Faculty of Pharmacy, University of Pécs, Rókus utca 2, H-7642 Pécs, Hungary; 5 Department of Food and Drug, University of Parma, Via G.P. 7 Usberti 17/A, 43124 Parma, Italy; luca.dellafiora@unipr.it (L.D.); chiara.dallasta@unipr.it (C.D.); 6 Department of Chemistry, Biology and Biotechnology, University of Perugia, Via Elce di Sotto 8, 06123 Perugia, Italy; gabri@chemiome.chm.unipg.it; 7 Department of Pharmacology and Pharmacotherapy, Medical School, University of Pécs, Szigeti út 12, H-7624 Pécs, Hungary

**Keywords:** alternariol, serum albumin, albumin-ligand interaction, binding site, fluorescence spectroscopy

## Abstract

Alternariol (AOH) is a mycotoxin produced by *Alternaria* species. In vitro studies suggest the genotoxic, mutagenic, and endocrine disruptor effects of AOH, and an increased incidence of esophageal cancer has been reported related to higher AOH exposure. Human serum albumin (HSA) is the most abundant plasma protein in the circulation, it is able to affect toxicokinetic properties of numerous xenobiotics. HSA forms stable complexes with several mycotoxins, however, the interaction of AOH with albumin has not been examined. In this study, the complex formation of AOH with HSA was tested, employing fluorescence spectroscopy, ultrafiltration, and molecular modeling. Each spectroscopic measurement shows the formation of stable AOH-HSA complexes (*K* = 4 × 10^5^ L/mol). Investigations with site markers (in spectroscopic and ultrafiltration models) as well as modeling studies suggest that AOH occupies Sudlow’s site I as a high-affinity binding site in HSA. The binding affinity of AOH towards bovine, porcine, and rat albumins was also tested, suggesting that AOH binds to rat albumin with considerably higher affinity than other albumins tested. Our results demonstrate the strong interaction of AOH with serum albumins, suggesting the potential in vivo importance of these interactions.

## 1. Introduction

**Figure 1:**
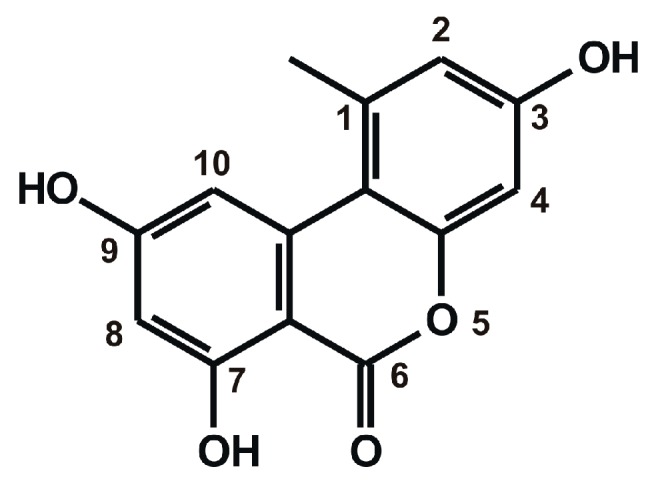
Chemical structure of alternariol.

Mycotoxins are secondary metabolites of filamentous fungi, exerting adverse effects in humans and animals [1,2]. *Alternaria* species contaminate unprocessed food (e.g., cereal crops, grape, tomato, oilseeds, and citrus fruits) [3,4,5] and processed products (e.g., apple juice, wine, and tomato paste) [6,7,8]. *Alternaria* molds are responsible for the post-harvest decay of agricultural products, even during refrigerated transport and storage, due to their ability to grow even at low temperature [9]. Alternariol (AOH; Figure 1) is a dibenzo-α-pyrone mycotoxin produced by *Alternaria* species, it shows structural similarity with known xenoestrogens such as urolithins and genistein [10]. The acute toxicity of AOH is considered to be low [11], and its chronic in vivo toxicity is also controversial. Increased incidence of esophageal cancer has been reported in some areas with higher AOH exposure [12,13]. Genotoxic, mutagenic, and endocrine disruptor effects of AOH have also been described in several in vitro studies. AOH inhibits the catalytic activity of topoisomerase I and II enzymes [5,14,15]. Furthermore, Tiessen et al. [16] demonstrated that AOH (as a pro-oxidant) can change the cellular redox status, however, no oxidative stress-related DNA damage was observed in cell culture. AOH has weak estrogenic and androgenic/antiandrogenic effects, however, the dietary exposure results in its relatively low plasma concentrations (5–10 nM) which makes questionable the in vivo relevance of the endocrine disruptor effects of AOH [10,11,17,18].

Human serum albumin (HSA) is the most abundant plasma protein in the human circulation. HSA maintains the oncotic pressure of blood regulating the volume of the intravascular space, as well as it has buffer, pseudo–enzymatic, and antioxidant functions [19]. Furthermore, HSA forms stable complexes with several endogenous and exogenous compounds, including ions, fatty acids, drug molecules, and toxic xenobiotics [19,20,21,22]. The formation of high-affinity HSA-ligand complexes can affect the membrane transport, distribution, and elimination of ligand molecules [23,24]. HSA contains three domains (I–III), each includes two subdomains (called A and B). Sudlow’s site I (SSI) and Sudlow’s site II (SSII) are well-known drug binding sites in HSA [19,25], however, recent studies revealed the importance of Heme binding site (or FA1) [26]. SSI is an apolar cavity in subdomain IIA which includes the sole tryptophan residue of HSA (Trp-214), SSII is located in subdomain IIIA, while the Heme site is an apolar pocket in subdomain IB [19]. Previous studies highlighted that several mycotoxins (e.g., ochratoxins, citrinin, and zearalenone) form highly stable complexes with HSA [27,28,29,30]. However, based on our current knowledge, the interaction of AOH with serum albumin has not yet been investigated. Since the albumin-binding of xenobiotics may be a relevant detail regarding their toxicokinetics, it is important to determine the affinity of formed complexes and identify their high-affinity binding site(s) on HSA.

In this study, the interaction of AOH with albumin was investigated. The binding constant (*K*) of AOH-HSA complex was quantified based on three spectroscopic methods, and binding site of AOH in HSA was determined by site markers using spectroscopic and ultrafiltration techniques. To get a deeper insight into the binding position of AOH, modeling studies were performed. Furthermore, species differences of AOH-albumin interaction were also tested with bovine (BSA), porcine (PSA), and rat (RSA) serum albumins. Our results demonstrate that AOH forms high-affinity complexes with HSA (*K* = 4 × 10^5^ L/mol), during which AOH occupies SSI as binding site. Stability of AOH-albumin complexes are similar (regarding HSA, BSA, and PSA), while AOH binds to RSA with significantly higher affinity compared to the other albumins tested.

## 2. Results

### 2.1. Fluorescence Spectra of AOH in the Absence and Presence of HSA

**Figure 2:**
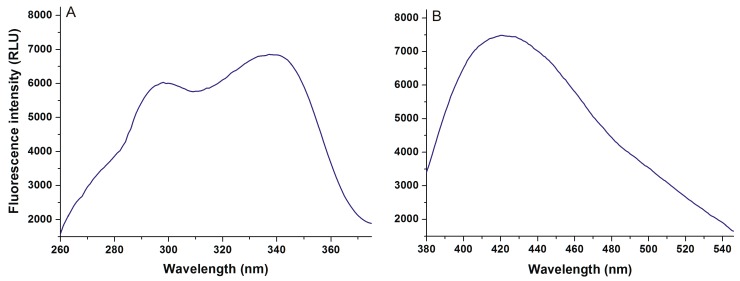
Fluorescence excitation (**A**) and emission (**B**) spectra of 50 μM alternariol (AOH) in phosphate buffered saline (PBS) (pH 7.4; λ_ex_ = 345 nm, λ_em_ = 421 nm).

**Figure 3:**
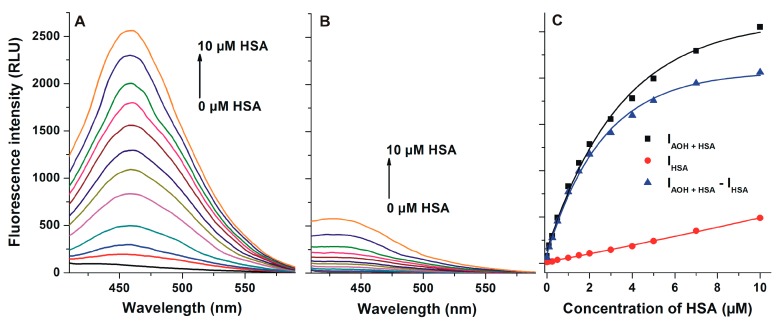
(**A**) Fluorescence emission spectrum of AOH (1 μM) in the presence of increasing human serum albumin (HSA) concentrations (0.0, 0.1, 0.25, 0.5, 1.0, 1.5, 2.0, 3.0, 4.0, 5.0, 7.0, and 10.0 μM) in PBS (pH 7.4); (**B**) Fluorescence emission spectrum of increasing concentrations of HSA (0.0, 0.1, 0.25, 0.5, 1.0, 1.5, 2.0, 3.0, 4.0, 5.0, 7.0, and 10.0 μM) in PBS; (**C**) Fluorescence emission intensity of AOH in the presence of increasing concentrations of HSA (I_AOH+HSA_), HSA alone (I_HSA_), and I_AOH+HSA_ – I_HSA_ (λ_ex_ = 345 nm, λ_em_ = 455 nm).

First, the fluorescence excitation and emission spectra of AOH were recorded in PBS (pH 7.4). AOH exerts fluorescence under physiological conditions (Figure 2), its excitation wavelength maximum was noticed at 345 nm (λ_em_ = 421 nm), while its emission maximum appeared at 421 nm (λ_ex_ = 345 nm). In the presence of HSA, a red shift (421 → 455 nm) of the fluorescence emission spectrum was noticed, and the fluorescence signal of AOH was markedly increased (Figure 3A). Under the applied circumstances, the HSA preparation (without AOH) also showed fluorescence at 455 nm (Figure 3B); however, the HSA-induced increase in the fluorescence of AOH was considerably higher than additive (I_AOH_ + I_HSA_) (Figure 3C).

### 2.2. Fluorescence Quenching Experiments

**Figure 4:**
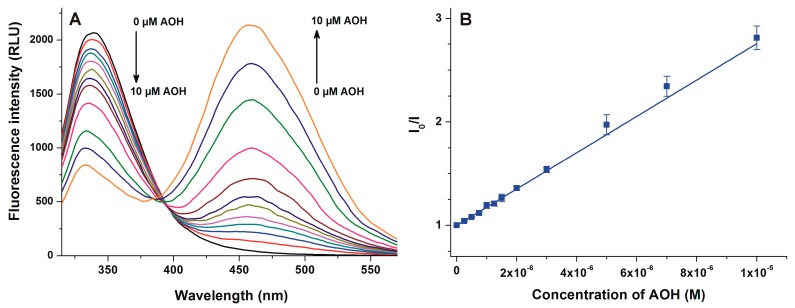
(**A**) Fluorescence quenching of HSA by AOH. Fluorescence emission spectrum of HSA (2 μM) in the presence of increasing concentrations of AOH (0.0, 0.25, 0.5, 0.75, 1.0, 1.25, 1.5, 2.0, 3.0, 5.0, 7.0, and 10.0 μM) in PBS (pH 7.4; λ_ex_ = 295 nm); (**B**) Stern-Volmer plot of AOH-HSA interaction (λ_ex_ = 295 nm, λ_em_ = 340 nm).

To further test the interaction of AOH with HSA, the quenching effect of AOH on the fluorescence of HSA was examined. To excite the Trp-214 residue of HSA, 295 nm was used as the excitation wavelength, resulting in its fluorescence emission peak at 340 nm [31]. Increasing concentrations of AOH induced a significant decrease in the fluorescence signal at 340 nm, showing a concentration-dependent fashion (Figure 4A). Furthermore, an increasing second peak appeared at 455 nm, which obviously resulted from the intrinsic fluorescence of the mycotoxin. Based on the decrease in the fluorescence signal of HSA in the presence of AOH, the Stern-Volmer equation was fitted to the experimental data. The Stern-Volmer plot shows good linearity (*R*^2^ = 0.996) and correlation with the 1:1 stoichiometry model (Figure 4B).

### 2.3. Interaction of AOH with HSA Based on Energy Transfer

**Figure 5:**
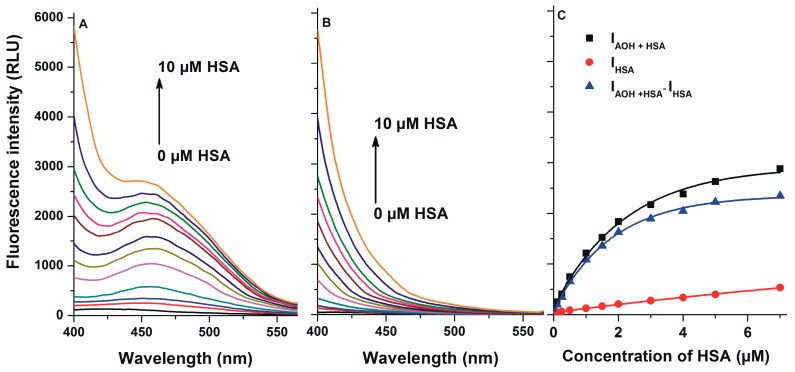
(**A**) Fluorescence emission spectrum of AOH (1 μM) in the presence of increasing concentrations of HSA (0.0, 0.1, 0.25, 0.5, 1.0, 1.5, 2.0, 3.0, 4.0, 5.0, 7.0, and 10.0 μM) in PBS (pH 7.4); (**B**) Fluorescence emission spectrum of increasing concentrations of HSA (0.0, 0.1, 0.25, 0.5, 1.0, 1.5, 2.0, 3.0, 4.0, 5.0, 7.0, and 10.0 μM) in PBS; (**C**) Fluorescence emission intensity of AOH in the presence of increasing concentrations of HSA (I_AOH+HSA_), HSA alone (I_HSA_), and I_AOH+HSA_ – I_HSA_ (λ_ex_ = 295 nm, λ_em_ = 455 nm).

In the following experiment, increasing concentrations of HSA were added to a standard amount of AOH (similarly to 2.1). However, emission spectra of these samples were recorded using the 295 nm excitation wavelength. This approach gives complex information because at 295 nm we excite the Trp-214 residue of HSA, and the emission maximum of HSA (340 nm), as well as the excitation maximum of AOH (345 nm), are very close. Therefore, the energy transfer between HSA and AOH may occur if the binding site of AOH is close to the Trp-214 moiety. Consequently, an increase in the fluorescence signal of AOH (at 455 nm) may appear despite the applied excitation wavelength is far from the excitation maximum of AOH. During these experiments, the emission signals of HSA (in the absence of AOH) were also recorded, to verify that the spectral changes are not only derived from the emission signal of the HSA preparation. As Figure 5 demonstrates, the fluorescence emission signal at 455 nm was markedly increased in the presence of HSA, and the increase in the fluorescence was considerably higher than the sum of AOH and HSA signals.

### 2.4. Stability of AOH-HSA Complex Based on Fluorescence Spectroscopic Measurements

The binding constant of AOH-HSA complex was determined based on the three fluorescence methods described in Section 2.1, Section 2.2 and Section 2.3, using Hyperquad2006 software. The non-linear fitting suggests the 1:1 stoichiometry of complex formation. Calculated decimal logarithmic values of binding constants (log*K*) and the Stern-Volmer quenching constant (log*K*_SV_) are demonstrated in Table 1. Binding constants determined by the three methods show good correlation and suggest the formation of stable AOH-HSA complexes.

### 2.5. Effects of Site Markers on the Fluorescence Signal of AOH-HSA Complex

**Figure 6:**
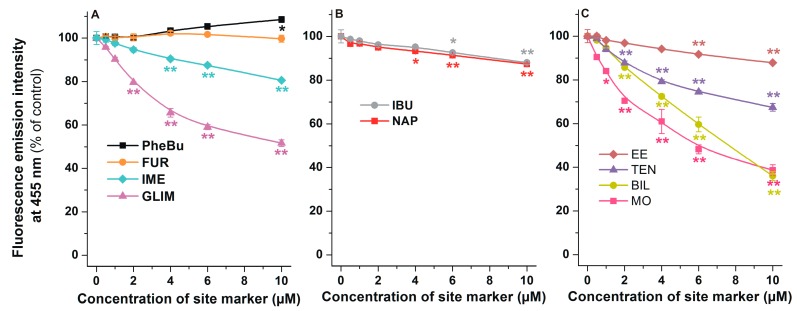
Fluorescence emission intensity of AOH (1 μM) in the presence of HSA (3 μM) and increasing concentrations (0.0, 0.5, 1.0, 2.0, 4.0, 6.0, and 10.0 μM) of SSI (**A**), SSII (**B**), and Heme site (**C**) markers in PBS (pH 7.4; λ_ex_ = 345 nm, λ_em_ = 455 nm; * *p* < 0.05, ** *p* < 0.01; PheBu, phenylbutazone; FUR, furosemide; GLIM, glimepiride; IME, indomethacin; NAP, naproxen; IBU, ibuprofen; MO, methyl orange; BIL, bilirubin; TEN, teniposide; EE, ethinylestradiol).

Since HSA induces a strong increase in the fluorescence of AOH (Figure 3), it is reasonable to hypothesize that the displacement of AOH from HSA leads to the significant decrease in the fluorescence signal at 455 nm (λ_ex_ = 345 nm). Using this principle, increasing concentrations of site markers of SSI, SSII, or Heme site were added to standard concentrations of AOH and HSA (1.0 and 3.0 μM, respectively), then fluorescence emission intensities were determined (λ_ex_ = 345 nm, λ_em_ = 455 nm). Among SSI markers tested, phenylbutazone and furosemide caused negligible or only slight changes in the fluorescence of the AOH-HSA complex; however, indomethacin and glimepiride induced considerable decreases (Figure 6A). Despite SSII markers naproxen and ibuprofen resulting in statistically significant decreases in fluorescence at 455 nm, these compounds did not produce large spectral changes (Figure 6B). Each ligand of the Heme site induced significant decrease in the fluorescence signal of AOH-HSA complex (Figure 6C). Ethinylestradiol caused slight, while teniposide, bilirubin, and methyl orange produced strong decrease in the fluorescence at 455 nm.

### 2.6. Displacement of Site Markers from HSA by AOH Based on Ultrafiltration Studies

**Figure 7:**
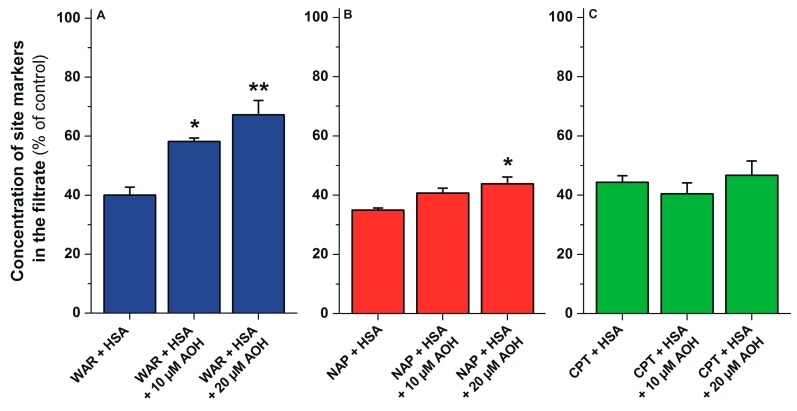
Displacement of Sudlow’s site I (SSI) (WAR, warfarin), Sudlow’s site II (SSII) (NAP, naproxen), and Heme site (CPT, S-camptothecin) ligands from HSA by AOH. Concentrations of warfarin (**A**), naproxen (**B**) and S-camptothecin (**C**) in the filtrates (% of control): Before the ultrafiltration, samples contained WAR and HSA (1.0 and 5.0 μM, respectively), NAP and HSA (1.0 and 1.5 μM, respectively), or CPT and HSA (1.0 and 1.5 μM, respectively) with or without 10 or 20 μM AOH in PBS (pH 7.4; * *p* < 0.05, ** *p* < 0.01). In each model, the filtered concentration of site markers was compared to the concentration measured in the filtrate when no HSA was added to the sample (100%).

To further test the involvement of SSI, SSII, and Heme sites regarding the interaction of AOH with HSA, ultrafiltration experiments were performed applying warfarin (SSI), naproxen (SSII), and S-camptothecin (Heme site) as site markers. In each assay, the same concentrations (1 μM) of site markers were used in the presence of the HSA concentration which, caused an approximately 60% decrease in the filtered site marker concentrations. After ultrafiltration, site markers were quantified by high-performance liquid chromatography (HPLC; see details in Section 4.4). Since HSA and HSA-bound site markers are unable to pass through the filter unit, increasing concentrations of site markers in the filtrate indicate their displacement from HSA. The filtered concentration of warfarin showed a concentration-dependent, marked increase in the presence of AOH, while AOH caused slight and no changes regarding the concentrations of naproxen and camptothecin in the filtrates, respectively (Figure 7).

### 2.7. Testing the Involvement of SSI and Heme Sites Based on Spectroscopic Studies

**Figure 8:**
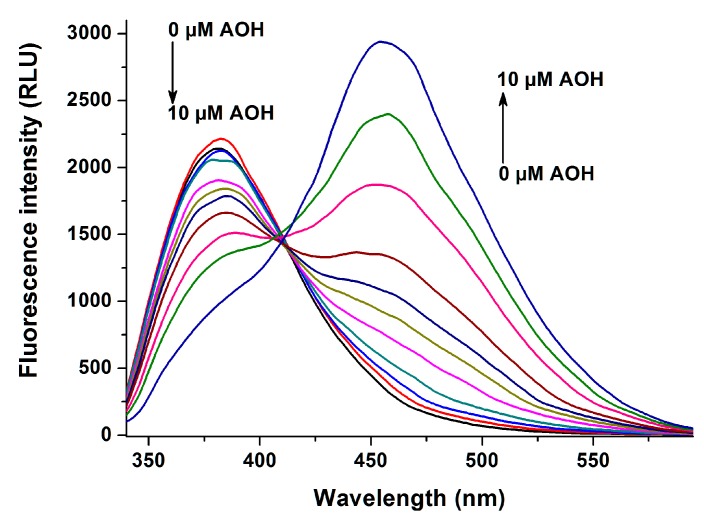
Fluorescence emission spectrum of warfarin (1.0 μM) in the presence of HSA (3.5 μM) and increasing concentrations of AOH (0.0, 0.1, 0.25, 0.5, 1.0, 1.5, 2.0, 3.0, 5.0, 7.0, and 10.0 μM) in PBS (pH 7.4; λ_ex_ = 317 nm).

The effect of AOH on warfarin-HSA interaction was also investigated employing the previously described method [29]. Since warfarin exerts significantly stronger fluorescence in HSA-bound form (vs. the unbound warfarin), the displacement of warfarin from HSA results in the strong decrease in fluorescence at 379 nm (λ_ex_ = 317 nm). Therefore, increasing concentrations of AOH were added to the warfarin-HSA complex, after which fluorescence emission spectra of these samples were recorded (see details in Section 4.2). AOH induced a concentration-dependent, strong decrease in the fluorescence at 379 nm (Figure 8). An increasing second peak also appeared at 455 nm, which is the emission signal of the mycotoxin.

In the following experiment, the effect of AOH on the absorbance of methyl orange-HSA complex was tested. During the interaction of methyl orange with HSA, its absorbance value increases as well as its absorption spectrum shows a red shift (464 → 477 nm) [30]. Therefore, it is reasonable to hypothesize that the displacement of methyl orange from HSA leads to the decrease in its absorption at 477 nm. However, AOH (even at three-fold concentration compared to methyl orange) did not affect the absorption spectrum of methyl orange-HSA complex (data not shown; see experimental details in 4.2).

### 2.8. Molecular Docking Studies

**Figure 9:**
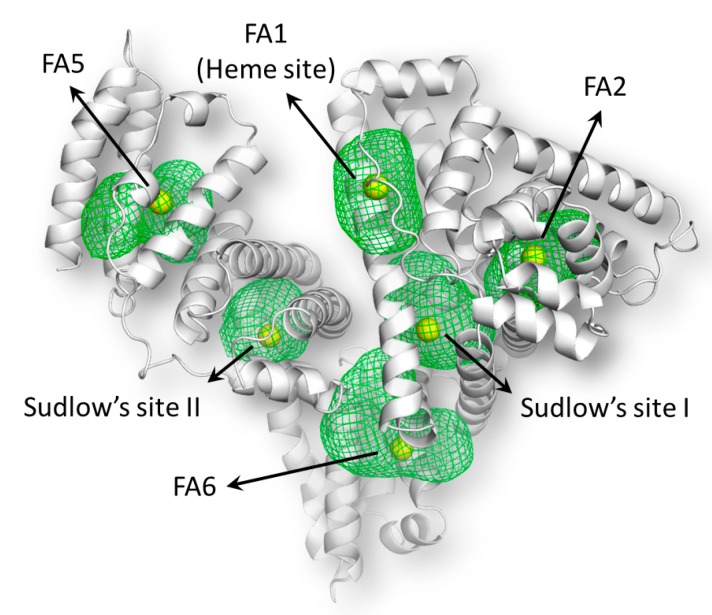
Graphical representation of binding sites mapping of HSA. The protein is represented by white cartoon while binding sites are represented by green mash. The yellow spheres indicate the centroids of binding sites.

In parallel with experimental studies, a molecular modeling study was also performed in order to map the binding site(s) of AOH in HSA as well as to define the binding conformation(s) of AOH in its binding site. After the various binding sites of HSA were mapped, one centroid for each site was set to define the space explored by docking simulations to dock AOH (Figure 9; see details in Section 4.5).

The independent docking simulations were performed in each site (Figure 9) to identify the binding site(s) involved in AOH-HSA interaction. After the collection of docking poses, a rescoring procedure using the HINT (Hydropathic INTeractions) scoring function was carried out. The reliability of this method was previously demonstrated as it succeeded to predict protein-ligand (including HSA-ligand) complex formation [32,33]. According to the rescoring procedure, AOH is possibly able to dock into FA1 (Heme site), SSI, and FA6 positions (Table 2).

**Figure 10:**
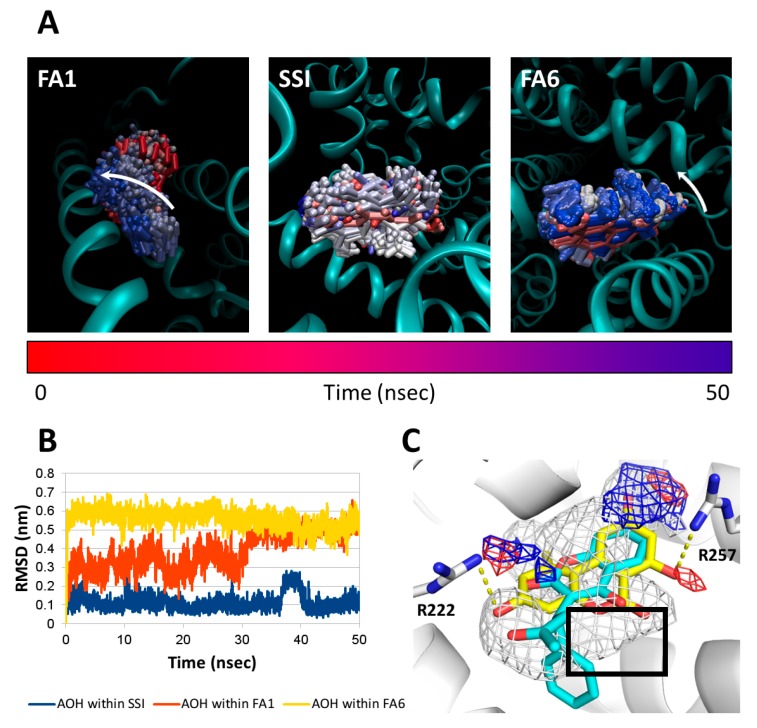
Results of molecular dynamic (MD) simulations and docking studies. (**A**) Time-step representation of the AOH trajectories within FA1 (Heme site), SSI, or FA6 sites. The from-red-to-blue color switch indicates the stepwise changes of AOH coordinates along the MD simulation. The trajectory outward the binding site within FA1 and FA6 is indicated by the white arrow; (**B**) root-mean-square analysis (RMSD) plot of AOH within FA1, SSI, or FA6 sites; (**C**) Calculated binding architecture of AOH (represented in yellow sticks) in comparison to the crystallographic pose of R-(+)-warfarin (represented by cyan sticks) (PDB (Protein Data Bank) code 1H9Z) [35]. The residues involved in polar interactions with AOH are represented by white sticks, while polar contacts are represented by yellow dotted lines. White, red, and blue meshes indicate regions sterically and energetically favorable to receive hydrophobic, hydrogen bond acceptor, and hydrogen bond donor groups, respectively. The black box indicates the pharmacophoric mismatch caused by the placing of the polar α-benzopyrone moiety of AOH within the hydrophobic space of the pocket.

After the identification of possible binding sites, molecular dynamic (MD) simulations were performed to monitor the capability of AOH to stably persist therein. Therefore, the trajectory of AOH and the root-mean-square analysis (RMSD) of its atomic coordinates along the MD simulation were analyzed to measure its structural stability within FA1, SSI, and FA6 sites. As it is demonstrated in Figure 10, AOH shows stable interaction with SSI (i.e., low and stable RMSD values were recorded). However, the interactions of AOH with FA1 (Heme site) and FA6 showed a higher instability retracing trajectories outward the respective binding site. In addition, the geometrical cluster analysis of AOH trajectory within SSI revealed a unique cluster, which further supports the stable interaction of AOH with the SSI binding site.

The capability of AOH to satisfy the pharmacophoric requirements of the SSI pocket was further investigated by comparing the midpoint pose of the cluster with the pharmacophoric fingerprint of SSI, also in comparison to the crystallographic pose of R-(+)-warfarin (taken as a reference for SSI ligands). AOH showed a binding pose which is markedly resembling the arrangement of the coumarin moiety of R-(+)-warfarin (Figure 10C). In particular, the polycyclic hydrophobic core of AOH was found satisfying the hydrophobic environment of the pocket, though the arrangement of the polar α-benzopyrone moiety within the hydrophobic environment was likely to cause degrees of interference. Nevertheless, the hydroxyl groups in positions 3 and 9 were found engaging in polar contact with Arg222 and Arg257, respectively.

### 2.9. Species Dependence of AOH-Albumin Interaction

**Figure 11:**
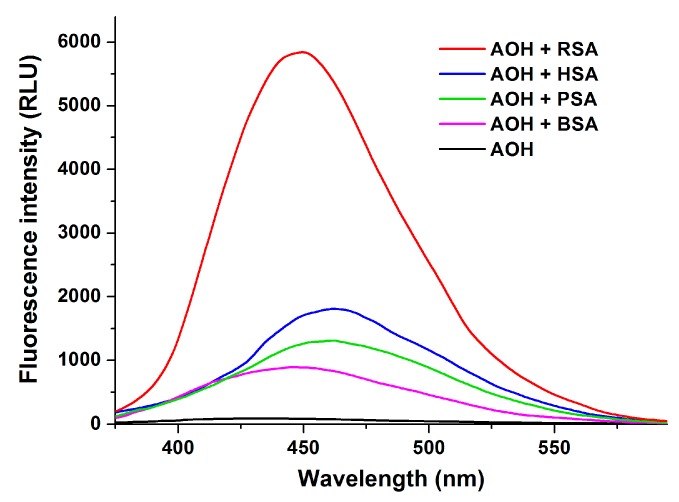
Fluorescence emission spectrum of AOH (1 μM) in the absence and presence of human (HSA), bovine (BSA), porcine (PSA), and rat (RSA) serum albumins (each 10 μM) in PBS (pH 7.4) (λ_ex_ = 345 nm; spectra were corrected with the background fluorescence of albumin preparations).

To test the potential species-dependent alternations of AOH-albumin interaction, the enhancement in the fluorescence signal of the mycotoxin by human, bovine, porcine, and rat serum albumins was investigated. Each albumin induced the strong elevation in the fluorescence emission signal of AOH; however, considerable differences in the extent of the enhancement were observed (Figure 11). RSA induced highly the strongest increase in the fluorescence of AOH (77-fold), followed by HSA (30-fold), PSA (20-fold), and BSA (14-fold) (λ_em_ = 455 nm). Based on the albumin-induced increase in the fluorescence of AOH (as demonstrated for AOH-HSA in Figure 3), binding constants of AOH-albumin complexes were determined by the Hyperquad2006 software. As Table 2 demonstrates, binding constants of AOH-HSA, AOH-BSA, and AOH-PSA are in the same range. Stability of AOH-HSA and AOH-PSA complexes are similar, while AOH binds to BSA with approximately two-fold higher affinity than to HSA or PSA. However, the stability of the AOH-RSA complex is considerably higher compared to the other AOH-albumin complexes tested, showing an approximately eight-fold higher binding constant vs. AOH-HSA (Table 3).

## 3. Discussion

AOH exerted only relatively low fluorescence at 455 nm without HSA; however, its fluorescence emission signal considerably increased in the presence of albumin (an approximately 30-fold increase was noticed with ten-fold HSA vs. AOH). Since the HSA-induced enhancement in the fluorescence highly exceeds the fluorescence signals of AOH and HSA (Figure 3), it is reasonable to hypothesize the formation of AOH-HSA complexes. Water molecules are commonly able to partly quench the fluorescence signal of aromatic fluorophores [36], therefore, the interaction of AOH with an apolar pocket in albumin leads to the partial decomposition of its hydration shell. Consequently, the decreased quenching effect of water molecules leads to the significant increase in the fluorescence signal of AOH. The complex formation of AOH with HSA was also confirmed by fluorescence quenching studies. After the correction of the inner-filter effect of AOH (Equation (1)), the mycotoxin strongly decreased the fluorescence of HSA at 340 nm, even at nanomolar concentrations (vs. 2 μM HSA; Figure 4). Under the applied circumstances, the fluorescence emission of HSA is mainly exerted by the Trp-214 residue [19], therefore, the strong quenching effect suggests that the binding site of AOH is located near to the Trp-214 moiety. This hypothesis is strongly supported by the energy transfer between HSA and AOH (Figure 5). Using 295 nm as the excitation wavelength, unbound AOH molecules exert negligible fluorescence emission at 455 nm. Previous studies have already demonstrated that fluorescence energy transfer may occur if the emission spectrum of the donor molecule (HSA) and the excitation spectrum of the acceptor (ligand) overlay, and there is a properly short distance between the fluorophores [37,38,39]. Thus, the selective excitation of albumin-bound AOH molecules can be explained by the following: (1) the emission spectrum of HSA and the excitation spectrum of AOH strongly overlap; (2) the binding site of AOH is very close to the Trp-214 amino acid (which is located in SSI) of HSA. Therefore, the energy transfer between HSA and AOH confirms not only the formation of AOH-HSA complexes, but gives information regarding the binding site of the mycotoxin in albumin, which is likely located in SSI.

The fact that the three applied fluorescence spectroscopic methods suggest very similar log*K* values validates the calculated binding constants (Table 1). Furthermore, each method suggests 1:1 stoichiometry of complex formation, which is in agreement with the evaluation based on the Stern-Volmer equation. Our results show that AOH forms stable complexes with HSA. According to previous studies with other mycotoxins, the binding constant of AOH-HSA is in the same range with citrinin-HSA and zearalenone-HSA complexes (10^5^ L/mol); however, the affinity of AOH towards HSA is approximately two-fold and three-fold higher vs. citrinin and zearalenone, respectively [29,40]. Furthermore, the binding constant of AOH-HSA is comparable with the warfarin-HSA complex (log*K* = 5.3) [29,41], suggesting the potential in vivo importance of the complex formation.

Since emission intensities were corrected based on the absorbance values of site markers (Equation (1)), the strong decreases in the fluorescence of the AOH-HSA complex in the presence of site markers suggest the displacement of the mycotoxin from albumin (Figure 6). Phenylbutazone and furosemide (bind to SSI and do not have secondary binding sites) [26] only slightly affected the fluorescence of the AOH-HSA complex. It is not surprising because SSI is a large region which makes possible the cooperative binding of ligands to the binding site [42,43]. However, the SSI markers likely possessing secondary binding sites in the Heme site (indomethacin and glimepiride) [26,44,45] strongly reduced the emission intensity of AOH-HSA. Based on these results, it is reasonable to hypothesize the potential involvement of SSI or Heme site regarding AOH-HSA interaction. However, this observation can also be explained by the fact that these two binding sites are allosterically coupled [19]. Methyl orange induced similar changes in the fluorescence of AOH-HSA complex than bilirubin (Figure 6). Since bilirubin binds to HSA with much higher affinity to HSA compared to methyl orange [26], it is unlikely that the interaction of these Heme site ligands with AOH is competitive.

In ultrafiltration studies, the displacement of SSI, SSII, and Heme site markers was tested. Since HSA is a macromolecule (with an approximately 67 kDa molecular weight), albumin and albumin-bound molecules cannot pass through the filter with 10 kDa molecular weight cut-off value; therefore, only unbound ligand molecules will appear in the filtrate [29]. AOH failed to considerably affect the concentrations of naproxen and S-camptothecin in the filtrates; however, the marked increase of filtered warfarin (Figure 7) supports the hypothesis that SSI is the high-affinity binding site of AOH in HSA.

Moreover, the fluorescence emission signal of the warfarin-HSA complex was strongly decreased by AOH (Figure 8), suggesting that the mycotoxin is able to displace the SSI marker warfarin from HSA (unbound warfarin molecules exert negligible fluorescence vs. albumin-bound warfarin) [29]. On the other hand, AOH failed to affect the absorbance of methyl orange-HSA complex, which makes again unlikely that the binding site of AOH is located in the Heme site [30]. Considering our previous observation that methyl orange can strongly decrease the fluorescence intensity of AOH-HSA complex (Figure 6), it is reasonable to hypothesize the allosteric interaction of Heme site ligands with AOH. This hypothesis is also compatible with the involvement of SSI as the high-affinity binding site of AOH.

The integration of computational data with the experimental results further supports SSI as the most likely binding site of AOH in HSA. Indeed, the in silico workflow pointed out the capability of AOH to preferentially interact and persist within the SSI site retracing the binding mode shown by the coumarin part of R-(+)-warfarin. The capability to match the pharmacophoric fingerprint of the SSI site was also highlighted, suggesting the interaction of AOH with SSI. Furthermore, it is worth noticing that the computational workflow presented here proved to be a reliable and straightforward strategy to scout binding sites in a multi-sites protein.

Most of the albumins examined (HSA, BSA, PSA) showed low species dependent alternations regarding AOH-albumin interactions; however, the binding constant of AOH-RSA complex highly exceeds the binding constants of other AOH-albumin complexes tested. The observed eight-fold difference in the affinity of AOH-RSA and AOH-HSA complexes may cause significant toxicokinetic differences between rat and man. Ochratoxin A-albumin and zearalenone/zearalenol-albumin complexes also showed similar extent of species-dependent variations in previous studies [40,46]. As it has been reported, the high species differences of ochratoxin A-albumin interactions significantly affect its toxicokinetics in different species [47].

In summary, the complex formation of AOH with albumin was investigated using fluorescence spectroscopic, ultrafiltration, and molecular modeling studies. Considering the high affinity of AOH towards HSA, it is reasonable to hypothesize the biologically relevant interaction. Spectroscopic, ultrafiltration, and modeling studies suggest that the binding site of AOH is located in SSI in HSA. However, both SSI and Heme site ligands can significantly displace the mycotoxin from albumin. Most albumins tested showed slight species differences, except the rat albumin which binds AOH with significantly higher affinity.

## 4. Materials and Methods

### 4.1. Reagents

Alternariol (AOH) was obtained from Cfm Oskar Tropitzsch (Marktredwitz, Germany). Racemic warfarin (WAR), naproxen (NAP), human serum albumin (HSA), bovine serum albumin (BSA), porcine serum albumin (PSA), rat serum albumin (RSA), glimepiride (GLIM), furosemide (FUR), bilirubin (BIL), phenylbutazone (PBut), indomethacin (IME), racemic ibuprofen (IBU) and S-camptothecin (CPT) were purchased from Sigma-Aldrich (Budapest, Hungary). Ethinylestradiol (EE) was purchased from Serva (Budapest, Hungary). Spectroscopic grade ethanol (96%), as well as HPLC-grade acetonitrile and methanol were obtained from VWR (Budapest, Hungary). Stock solutions of AOH (5000 μM), bilirubin (500 μM), methyl orange (2000 μM), and glimepiride (2000 μM) were prepared in spectroscopic grade dimethyl sulfoxide (DMSO; Fluka, Bucharest, Romania). Stock solutions of indomethacin and ethinylestradiol (both 2000 μM), as well as ibuprofen, furosemide, phenylbutazone, and naproxen (each 2500 μM) were prepared in ethanol (96%, spectroscopic grade). The applied amounts of organic solvents did not affect significantly the fluorescence measurements (tested in each spectroscopic model). All stock solutions were stored at −20 °C, and protected from light.

### 4.2. Spectroscopic Studies

Fluorescence spectra were recorded in the presence of air at 25 °C, employing a Hitachi F-4500 fluorescence spectrophotometer (Tokyo, Japan). The medium was phosphate buffered saline (PBS, pH 7.4) to approximate extracellular physiological conditions.

Binding constant of AOH-HSA complex was determined applying three fluorescence-based methods. Fluorescence quenching studies were carried out in the presence of HSA (2 μM) and increasing concentrations of AOH (0.0, 0.25, 0.5, 0.75, 1.0, 1.25, 1.5, 2.0, 3.0, 5.0, 7.0, and 10.0 μM), using 295 and 340 nm as excitation and emission wavelengths, respectively. Quenching experiments were evaluated using the graphical application of the Stern-Volmer equation and employing non-linear fitting by the Hyperquad2006 software [48] as described [39,40].

Thereafter, increasing amounts of albumin (final concentrations: 0.0, 0.1, 0.25, 0.5, 1.0, 1.5, 2.0, 3.0, 4.0, 5.0, 7.0, and 10.0 μM) were added to AOH (1 μM). Then, fluorescence emission spectra of these samples were recorded using 295 and 345 nm as excitation wavelengths, during which the Trp-214 residue of HSA and the AOH molecule was excited, respectively. The binding constant of AOH-albumin complexes were determined employing the Hyperquad2006 software [48] as described [39,40].

The effects of different site markers on the albumin-binding of AOH were also tested. Several markers of SSI (phenylbutazone, furosemide, glimepiride, and indomethacin), SSII (naproxen and ibuprofen), and Heme site (methyl orange, bilirubin, teniposide, and ethinylestradiol) were applied. Increasing amounts of site markers (final concentrations: 0.0, 0.5, 1.0, 2.0, 4.0, 6.0, and 10.0 μM) were added to AOH and HSA (1.0 and 3.0 μM, respectively) in PBS (pH 7.4). Under the applied conditions, solvents (DMSO or ethanol) did not significantly affect the fluorescence emission of the AOH-HSA complex.

To investigate the displacement of the SSI marker warfarin from HSA by AOH, the previously-reported fluorescence spectroscopic method was applied [30]. Fluorescence emission spectrum of warfarin (1.0 μM) was recorded in the presence of HSA (3.5 μM) and increasing concentrations of AOH (0.0, 0.1, 0.25, 0.5, 1.0, 1.5, 2.0, 3.0, 5.0, 7.0, and 10.0 μM) in PBS (pH 7.4; λ_ex_ = 317 nm).

To eliminate the potential inner-filter effect of test compounds (including AOH and site markers), absorption spectra were recorded using a Dynamica Halo DB-20 UV-Vis spectrophotometer (Dietikon, Switzerland), after which fluorescence data were corrected according to the following equation [49]:

I_corr_ = I_obs_ × e^(A^_ex_^+ A^_em_^)/2^ (1)

where *I*_corr_ and *I*_obs_ denote the corrected and the measured fluorescence emission intensity, respectively; while *A*_ex_ and *A*_em_ are the absorption of the compound at the excitation and emission wavelengths used, respectively.

Methyl orange has been reported to bind to HSA in subdomain IB (Heme site) [26]. To test the involvement of the Heme site regarding AOH-HSA interaction, the effect of AOH on the absorption spectrum of methyl orange-HSA complex was investigated, based on our previously described method [30]. Increasing amounts of AOH (final concentrations: 0, 2.5, 5, 10, 15, 20, and 30 μM) were added to methyl orange and HSA (10 and 15 μM, respectively) in PBS, then UV-Vis spectra of samples were recorded.

### 4.3. Ultrafiltration

To test further the binding site of AOH in HSA, ultrafiltration experiments were carried out with markers of SSI (warfarin), SSII (naproxen), and Heme site (S-camptothecin, CPT). Ultrafiltration studies with warfarin and naproxen were performed as described [50]. Briefly, Pall Microsep™ Advance centrifugal devices were used with 10 kDa molecular weight cut-off value. Before the ultrafiltration, filter units were rinsed once with 3.0 mL distilled water and twice with 3.0 mL PBS. Samples contained warfarin and HSA (1.0 and 5.0 μM, respectively), naproxen and HSA (1.0 and 1.5 μM, respectively), or CPT and HSA (1.0 and 1.5 μM, respectively) with or without 10 or 20 μM of AOH in PBS. Samples were centrifuged for 10 min at 7500 *g* and 25 °C (fixed angle rotor). Filtrates were analyzed with HPLC-FLD or HPLC-UV methods (see details in Section 4.4). Warfarin and naproxen were directly analyzed from the filtrates; however, in order to prevent the spontaneous conversion of camptothecin to an open-chain carboxylate form [51], the following sample preparation was performed. A 500-μL aliquot of filtrates was acidified with 2 μL of 6 M perchloric acid, and after a four-fold dilution with the HPLC mobile phase (see in Section 4.4), camptothecin was analyzed by HPLC.

To validate our new ultrafiltration model which aims to investigate the Heme site, the effects of two known site markers (methyl orange and bilirubin) were tested. Both methyl orange and bilirubin were able to significantly increase the concentration of CPT in the filtrate (Figure S1), suggesting the displacement of CPT from HSA. Furthermore, bilirubin showed much stronger effect than methyl orange, which is in agreement with the significantly higher binding affinity of bilirubin towards HSA (compared to methyl orange and CPT) [26].

### 4.4. HPLC Analyses

Warfarin and naproxen were quantified as previously described without any modifications [50]. The integrated HPLC system was built up from a pump (Waters 510), an injector (Rheodyne 7125) with a 20 μL sample loop, an UV-detector (Waters 486) and a fluorescent detector (Jasco FP-920). Chromatographic data were evaluated with Millennium Chromatography Manager software (Milford, MA, US). Briefly, samples containing warfarin were driven through a NovaPak C18 (4.0 × 3.0 mm) guard cartridge linked to a NovaPak C18 (150 × 3.9 mm, 4.0 μm) analytical column. Sodium phosphate buffer (25 mM, pH 7.0), methanol, and acetonitrile (70:25:5 *v*/*v*%) was applied as a mobile phase. The isocratic elution was performed with 1.0 mL/min flow rate. Warfarin was quantified by fluorescence detector (λ_ex_ = 310 nm, λ_em_ = 390 nm). Samples containing naproxen were driven through a Phenomenex Security Guard™ cartridge (C18, 4.0 × 3.0 mm) coupled to a Phenomenex Gemini C18 (150 × 4.6 mm, 3.0 μm) analytical column. The mobile phase was composed of sodium acetate buffer (6.9 mM, pH 4.0) and acetonitrile (50:50 *v*/*v*%). The isocratic elution was performed with 1.0 mL/min flow rate. Naproxen was quantified based on its absorbance at 230 nm.

S-camptothecin content of filtrates was determined employing the HPLC system (Jasco) contained a binary pump (PU-4180), an autosampler (AS-4050), and a FP-920 fluorescence detector. Chromatographic data were evaluated with ChromNAV software (Ver.2). Samples were driven through a Phenomenex Security Guard^™^ cartridge (C18, 4.0 × 3.0 mm) linked to a Teknokroma Brisa LC-2 C18 (150 × 4.6 mm, 3 μm) analytical column. The mobile phase consisted of orthophosphoric acid (10 mM) and acetonitrile (60:40 *v*/*v*%). The isocratic elution was performed with 0.8 mL/min flow rate. Camptothecin was quantified based in its fluorescence (λ_ex_ = 369 nm, λ_em_ = 437 nm).

### 4.5. Molecular Docking Studies

The molecular modeling study aimed at describing the interaction between AOH and HSA. The study used binding sites mapping, docking studies, pharmacophore modeling, and molecular dynamic simulations.

#### 4.5.1. Model Preparation

The 3D HSA model used in this study derived from the high-resolution crystallographic structure deposited in the Protein DataBank (https://www.rcsb.org) having PDB code 4L8U [24]. HSA structure in the presence of fatty acids was selected to keep the simulations close to the in vivo conditions. The structure of AOH was retrieved from PubChem Compound Database (https://pubchem.ncbi.nlm.nih.gov; CID = 5359485). Before proceeding with calculations, the consistency of atom and bond types of protein and AOH was checked with the Sybyl software (version 8.1; www.certara.com), as previously reported [33]. All sets of co-crystallized small molecules occupying the binding sites were removed to prepare the model for docking simulations.

#### 4.5.2. Binding Sites Mapping and Pharmacophoric Modeling

The mapping of binding sites aimed to define the localization of pockets within the HSA structure, and to define the space sterically available to arrange ligands. The pharmacophoric modeling aimed to describe the physicochemical properties of binding sites in terms of distribution of hydrophobic and hydrophilic features. The binding site mapping was performed using the Flapsite tool of the FLAP software (Fingerprint for Ligand And Protein; https://www.moldiscovery.com) while the GRID algorithm was used to investigate the corresponding pharmacophoric space [52,53]. The DRY probe was used to describe the potential hydrophobic interactions, while the sp2 carbonyl oxygen (O) and the neutral flat amino (N1) probes were used to describe the hydrogen bond acceptor and donor capacity of the target, respectively.

#### 4.5.3. Docking Study and Rescoring Procedure

Once we mapped the HSA pockets and defined the pocket centroids, the docking study aimed to assess the capability of AOH to arrange therein. The GOLD software [54] was used to perform all of the docking simulations, while a rescoring procedure using the HINT scoring function [55] was performed for the better evaluation of the protein-ligand interaction. HINT score relates to the free energy of binding (the higher the score means the stronger the interaction, while negative scores indicate the lack of appreciable interaction), it was proved to be reliable during the assessment of protein-ligand (including HSA-ligand) interactions [32,56]. The occupancy of binding sites was rewarded setting a 10 Å sphere at the respective pocket centroid. Software setting and docking protocol were used as reported [33].

#### 4.5.4. Molecular Dynamic Simulations

Molecular dynamic simulations were performed to study the dynamic of AOH-HSA interactions over time. All the positive AOH-pocket interactions (namely, the interaction with SSI, FA1 and FA6 sites) independently underwent molecular dynamic simulations over 50 nanosecond to assess the capability of AOH to persist into the different pockets during the considered timeframe. The best scored pose of AOH in each binding site was selected as the starting coordinate for molecular dynamic simulations. In all sets of molecular dynamic simulations, the sites not occupied by AOH were computed in the fatty acid-bond state. The coordinates of fatty acids reported in the PDB structure having 4L8U [24] were used. As an exception, the fatty acid occupancy of the Heme site, which is not in the bound state with a fatty acid in the 4L8U structure, was set on the basis of the fatty acid coordinates reported in the HSA structure having PDB code 1H9Z [35]. Molecular dynamic simulations were performed using GROMACS (version 5.1.4) [57] with CHARMM27 all-atom force field parameters support [58]. Each ligand has been processed and parameterized with CHARMM27 all-atom force field using the SwissParam tool [59]. Protein-ligand complexes were solvated with SPCE waters in a cubic periodic boundary condition, and counter ions (Na^+^ and Cl^−^) were added to neutralize the system. Prior to molecular dynamic simulation, the systems were energetically minimized to avoid steric clashes and to correct improper geometries using the steepest descent algorithm with a maximum of 5000 steps. Afterwards, all the systems underwent isothermal (300 K, coupling time 2 picosecond and isobaric (1 bar, coupling time 2 picosecond) 100 picosecond simulations before running 50 nanosecond simulations (300 K with a coupling time of 0.1 picosecond and 1 bar with a coupling time of 2.0 picosecond). The geometric cluster analysis was performed to sample similar AOH poses (within 1 Å RMSD) using the clustering algorithm described by Daura and coworkers [60], which was implemented in the gromos tool of GROMACS.

### 4.6. Statistics

In our plotted graphs, means and standard error of the mean (± SEM) were indicated, derived from at least three independent experiments. Statistical evaluation of data was performed employing IBM SPSS Statistics software, during which one-way ANOVA analysis was carried out with *p* < 0.05 and *p* < 0.01 levels of significance.

## Figures and Tables

**Table 1:** Decimal logarithmic values of Stern-Volmer quenching constant (*K*_SV_; unit: L/mol) and binding constants (*K*; unit: L/mol) of AOH-HSA complex. Data represent mean ± SEM (*n* = 3).

log*K_SV_* (SV-plot; Figure 4) λ_ex_ = 295 nm, λ_em_ = 340 nm	log*K* (Hyperquad; Figure 4) λ_ex_ = 295 nm, λ_em_ = 340 nm	log*K* (Hyperquad; Figure 5) λ_ex_ = 295 nm, λ_em_ = 455 nm	log*K* (Hyperquad; Figure 3) λ_ex_ = 345 nm, λ_em_ = 455 nm
5.31 ± 0.03	5.60 ± 0.01	5.78 ± 0.02	5.64 ± 0.05

**Table 2:** Interaction scores between AOH and the different binding sites in HSA.

Binding Sites	HINT Score	Expected Interaction
FA1 (Heme site)	852.44	Positive
FA2	−127.25	Negative
FA5	−356.23	Negative
FA6	160.34	Positive
Sudlow’s site I (SSI)	399.35	Positive
Sudlow’s site II (SSII)	−91.69	Negative

Note: The higher the score, the stronger the interaction, while negative scores indicate the lack of appreciable interactions [34].

**Table 3:** Decimal logarithmic values of binding constants (*K*; unit: L/mol) of AOH-albumin complexes. Data represent means ± SEM (*n* = 3).

Complexes	AOH-HSA	AOH-BSA	AOH-PSA	AOH-RSA
log*K*	5.64 ± 0.05	5.91 ± 0.04	5.61 ± 0.04	6.53 ± 0.01

## Supplementary Materials

Supplementary materials can be found at https://www.mdpi.com/1422-0067/20/9/2352/s1.

## Abbreviations

AOH	Alternariol
BSA	Bovine Serum Albumin
FLD	Fluorescence Detector
HINT	Hydropathic INTeractions
HPLC	High-Performance Liquid Chromatography
HSA	Human Serum Albumin
MD	Molecular dynamic
PDB	Protein Data Bank
PSA	Porcine Serum Albumin
RMSD	Root-Mean-Square Analysis
RSA	Rat Serum Albumin
SSI	Sudlow’s Site I
SSII	Sudlow’s Site II
