# Supplementary material for: Interaction of Mycotoxin Alternariol with Serum Albumin
Source: Int J Mol Sci. 2019 May 12;20(9):2352. doi: 10.3390/ijms20092352 (PMC6539399; doi:10.3390/ijms20092352)
Supplement: Supplementary file 1 [file ijms-20-02352-s001.pdf]

International Journal of
Molecular Sciences

Supplementary Materials

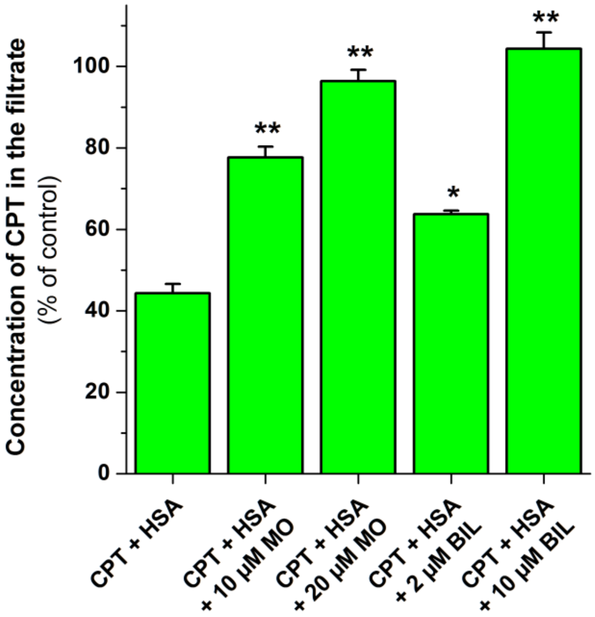

Figure S1. Displacement of S-camptothecin (CPT) from HSA by methyl orange (MO) and bilirubin (BIL). Concentration of CPT in the filtrate (% of control): Before the ultrafiltration, samples contained CPT and HSA (1.0 and 1.5 μM, respectively) with or without MO (10 and 20 μM) or BIL (2 and 10 μM) in PBS (pH 7.4; * $p < 0.05$, ** $p < 0.01$). The filtered concentration of CPT was compared to the concentration measured in the filtrate when no HSA was added to the sample (100%).
